# The Effect of Non-Uniform Material Distribution on the Bending Strength and Energy Absorption of TPMS Structures

**DOI:** 10.3390/polym18040455

**Published:** 2026-02-11

**Authors:** Martin Koroľ, Monika Töröková, Marek Kočiško

**Affiliations:** Faculty of Manufacturing Technologies, Technical University of Kosice, Bayerova 1, 08001 Presov, Slovakia; martin.korol@tuke.sk

**Keywords:** TPMS structures, additive manufacturing, gradient materials, bending tests, ductility index

## Abstract

Optimizing the mechanical response of structures with triple periodic minimal surfaces (TPMS) is key to their use in lightweight applications focused on energy absorption. This study evaluated the influence of cell geometry and uneven material distribution on the bending behavior of Primitive, Gyroid, and Diamond structures. Nylon 12 CF samples were produced using an additive method (FDM) with volume fractions of 35%, 40%, 45%, and 55%. The mechanical response was quantified using a three-point bending test according to ISO 178, from which the maximum force (F_max_), flexural strength (σ_f_), absorbed energy (E_abs_), and ductility index (µ_d_) were determined. The Primitive structure achieved the highest strength at a volume fraction of 45% (σ_f_ = 28.35 MPa; F_max_ = 756 N). The Primitive structure also demonstrated the highest toughness with a ductility index of up to µ_d_ = 8.62 at 55%. The study identified a significant deformation phenomenon in the Gyroid structure, where the sample with a volume fraction of 45% showed higher absorbed energy (34.58 J) than the sample with a higher fraction of 55% (26.81 J). This finding suggests that targeted material inhomogeneity (gradient) can, under specific conditions, lead to stabilization of the deformation mechanism through progressive collapse, thereby increasing energy efficiency. The Primitive structure proved to be the most resistant to uneven material distribution and, with a volume fraction of 45–55%, offers an optimal compromise between high strength and toughness, making it most suitable for the design of gradient structures subjected to bending loads.

## 1. Introduction

Three-dimensional periodic minimal surfaces (TPMS) have been intensively studied in recent years due to their exceptional ratio between weight, stiffness, and energy absorption [1,2]. They are characterized by a continuous, smooth topology without sharp transitions and without closed cavities, which makes them ideal for use in areas where effective stress flow control and controlled deformation are required. TPMS structures are based on periodic minimal surfaces, whose definition was originally formulated by Schoen [3]. The mechanical characteristics of various TPMS topologies—most commonly Schwarz Diamond, Schoen Gyroid, and Schwarz Primitive—have been analyzed in detail in several experiments and numerical studies.

Maskery et al. [4] demonstrated that TPMS structures show significant potential in terms of energy absorption, with their behavior strongly dependent on their volume fraction and surface orientation. Blanquer et al. [5] showed that Diamond and Gyroid achieve a significantly better stiffness-to-weight ratio than conventional lattice structures at higher densities. Current research is increasingly focused on analytical modeling of these structures, with Ma et al. [6] emphasizing that Diamond and Gyroid topologies exhibit bending-dominated behavior, which provides them with greater stability under gradual loading compared to the Primitive structure, which is prone to sudden failure due to stress localization. A review study by Yang et al. [7] identified TPMS as one of the most effective architectures for optimizing mechanical properties due to continuous stress transfer. Re, D. et al. [8] analyzed their fatigue life, and Attarzadeh et al. [9] demonstrated their transport efficiency in heat transfer.

There are several studies in the literature confirming that the mechanical behavior of TPMS structures can be effectively influenced by parameters such as material volume fraction, topology type, construction direction, or manufacturing process. However, most studies focus on structures with uniform material distribution, i.e., with constant wall thickness. Despite the theoretical advantages, however, there is a lack of in-depth experimental comparison in the literature of how uneven material distribution changes the deformation modes of these three basic topologies. However, as noted by Nguyen-Xuan et al. [10], uniform structures often fail to fully exploit the potential of the material under conditions of non-homogeneous stress fields, such as bending. This is where the concept of functionally graded materials (FGMs) comes in, allowing for spatial variation in mechanical properties. Zhang et al. [11] point out in this regard that uniform grids often fail due to the localization of shear bands at an angle of 45°, which can be prevented by introducing a functional gradient.

Yu et al. [12], Wang et al. [13], and Al-Ketan et al. [14] have shown that gradient TPMS can achievISOe higher elasticity and stability under deformation. However, most of this knowledge is based on simulations or pressure tests, and the behavior of gradient TPMS under complex bending loads remains poorly documented. More recent experimental studies, such as Gohar et al. [15], suggest that in three-point bending, the density gradient can effectively copy the bending moment curve, thereby increasing the overall stiffness of the beam without increasing its total weight. Although Villani et al. [16] and Ni et al. [17] confirmed that there are significant differences between Diamond, Gyroid, and Primitive in bending, the influence of the spatial gradient on the displacement of deformation zones in three-point bending has not yet been experimentally verified.

This problem is further intensified when using advanced composites. In the FDM process, strong fiber alignment occurs in thin TPMS walls [18,19]. Nylon 12 CF (PA12 CF) has a high modulus of elasticity and strength [20,21], but at the same time it is characterized by “brittle” fracture without a significant plastic region [22]. According to a study by Fang et al. [23], adding carbon fibers to a polymer matrix significantly increases the anisotropy of TPMS structures, which can lead to premature delamination of layers under complex loading. Previous research has largely ignored the combination of a highly rigid, brittle composite matrix (PA-12 CF) and gradient TPMS geometry. It is not known whether the wall thickness gradient can compensate for the brittleness of the material and increase the overall toughness of these structures under bending.

Given these gaps in knowledge, the aim of this study is to experimentally analyze the influence of linear gradient material distribution on the bending behavior of three TPMS topologies—Schwarz Diamond, Schoen Gyroid, and Schwarz Primitive—manufactured using FDM technology from Nylon 12 CF composite. The mechanical response was evaluated in terms of maximum force (F_max_), energy absorption (E_abs_), and ductility index (µ_d_) to assess whether gradient material distribution is an effective means of optimizing TPMS structures.

## 2. Materials and Methods

### 2.1. Design of TPMS Structured

In this study, three commonly used TPMS topologies—Schwarz Diamond, Schoen Gyroid, and Schwarz Primitive—were analyzed, representing three distinct morphological groups of minimal surfaces. These structures were generated using Creo Parametric 10 software (PTC Inc., Boston, MA, USA) and designed with variable material volume fractions according to the values listed in Table 1. Each of the topologies exhibits a specific geometric arrangement of surfaces, which affects their stiffness, deformation stability, and failure mechanism.

**Figure 1:**
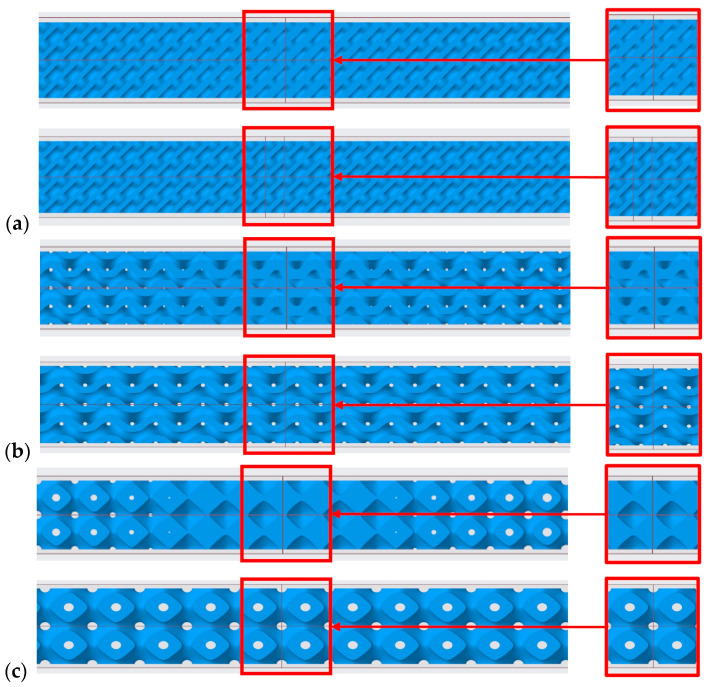
Illustration of TPMS structures and their comparison Gradient vs. Uniform (**a**) Diamond (**b**) Gyroid (**c**) Primitive.

The wall thickness in these models was designed to gradually thicken from both ends toward the center of the model in order to control the distribution of stress. Although several previous works [24] have focused on uniform configurations that exhibit predictable and homogeneous stiffness, the gradient architecture allows for controlled local strengthening or softening of the structure. This non-uniform distribution of material is key to targeted changes in the location and mechanism of failure, in contrast to traditional, predictable failure. The difference in architecture is demonstrated in cross-sections (Figure 1).

The mathematical representation of TPMSs is based on implicit equations of minimal surfaces. For the studied topologies, their shape can be briefly expressed as follows (see Table 1):

The constant *t* determines the shift in the isosurface and allows the resulting volume fraction of the material to be adjusted. The model itself was then converted into a solid with a defined wall thickness, with gradient variants generated using parametric functions determining the gradual change in thickness in a defined direction.

The target volume fractions of 35%, 40%, 45% and 55% were defined at the CAD model level as average values for the entire sample volume. These values were achieved by combining the isosurface parameter setting t and subsequent conversion of the surface model into a solid with a defined (locally variable) wall thickness. The volume fractions given therefore represent CAD-defined (nominal) values, not directly measured local densities of the printed samples.

CAD models for research were generated using Creo Parametric 10 software (PTC Inc., Boston, MA, USA). This software features a Lattice function that replaces full-volume material with a selected TPMS structure.

**Figure 2:**
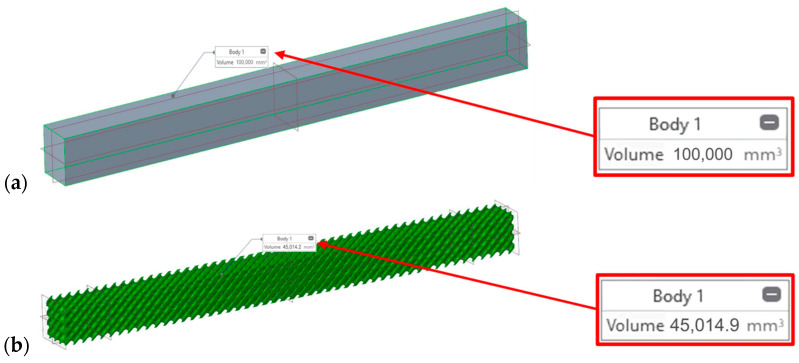
Before replacement with selected TPMS structure (**a**); after replacement with selected TPMS structure (**b**).

The samples for the experiment were designed as prisms with nominal dimensions of 20 × 20 × 250 mm. The internal volume was filled with three types of TPMS structures (Primitive, Gyroid, and Diamond) with a constant unit cell size of 10 × 10 × 10 mm. The main objective of the design was to introduce a symmetric linear density gradient that smoothly changes the stiffness of the sample along its length. (Figure 2).

Four types of samples were created, where the volume of the material present was gradually reduced to 35%, 40%, 45%, and 55% of the total volume. Three samples were created from each type to determine repeatability. This applied to all three selected TPMS structures (Diamond, Gyroid, and Primitive).

**Figure 3:**
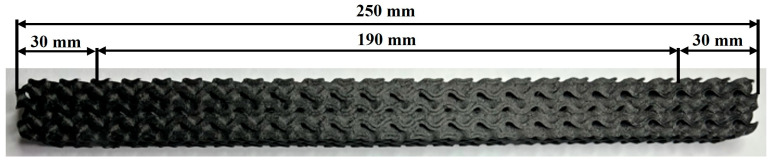
Display of structure densification within the creation of a software model on a real printed sample.

A key aspect of the design was the division of the sample into three functional zones. The outer zones, extending 30 mm from both ends of the sample, were defined with minimum local density. Towards the central part (middle 190 mm), the density increased linearly until it reached its maximum at the geometric center of the sample (125 mm from the edge). This design resulted in the creation of four types of samples for each topology, which differed in the total (average) volume fraction of material: 35%, 40%, 45%, and 55%. (Figure 3). The density of the material in the center of the sample increased based on an increase in the total percentage from 35% to 55%.

The material gradient was implemented directly in the CAD environment by locally adjusting the wall thickness of the TPMS along the length of the sample. No analytical thickness function was prescribed. Instead, a software gradient tool (PTC Creo Parametric 10) was used to gradually increase the wall thickness, starting at a distance of 30 mm from both ends of the sample towards the center. The thickness transition was applied symmetrically from both ends, resulting in a monotonic increase in local relative density towards the area exposed to the highest bending stresses.

The local density in the extreme zones (30 mm from both ends) was set at a constant value of 30% for all sample types. Towards the center of the sample, the density increased linearly so that the resulting total volume fractions reached values of 35%, 40%, 45%, and 55%. Three samples were produced from each combination of topology and density to ensure the statistical repeatability of the experiment. The total volume fraction given for each variant (35–55%) thus represents the average value of the integrated local density along the entire length of the sample.

#### Additive Manufacturing Parameters

To ensure reproducibility of the proposed gradient TPMS structures, all samples were manufactured using identical FDM process parameters, which are summarized in Table 2. Although micro-CT analysis was not used, print fidelity was verified by thorough visual inspection of all samples prior to testing. The print parameters were pre-calibrated on PA12-CF test cubes to ensure that the line width (0.45 mm) accurately matched the nominal wall thickness of the TPMS structures. The average dimensional deviation of the outer dimensions of the samples did not exceed ±0.15 mm, confirming the integrity of the FDM process. In addition, the weight of the printed samples was verified, with an average deviation from the theoretical weight of the CAD models of less than 3%, demonstrating the high density and accuracy of the internal material distribution without significant internal porosity.

These parameters were kept constant for all samples in order to isolate the influence of geometry and material distribution on the mechanical response.

### 2.2. Material Used—Nylon 12 CF (PA-12 CF)

PA 12 CF (MakerBot, New York, NY, USA) reinforced with carbon fibers is a composite thermoplastic that has gained significant application in additive manufacturing, particularly in fused deposition modeling (FDM) technology [25,26]. This material combines the toughness of polyamide 12 (PA12) with the high strength and stiffness of short chopped carbon fibers (typically 35% by weight), achieving the highest stiffness-to-weight ratio among most FDM thermoplastics [25,27]. The presence of carbon fibers significantly improves the structural characteristics of the material, making it an ideal replacement for metal components in specific cases, especially in the production of functional prototypes, fixtures, and tools [25,27]. As a result of additive manufacturing (FDM), the final product has anisotropic mechanical properties that depend on the orientation of the fibers and the direction of layer deposition, which is a key factor in the design of loaded parts [25,28].

The following typical values of mechanical properties (see Table 3) were determined according to the relevant ASTM (American Society for Testing and Materials) standards for samples produced using FDM technology [26]. These values are indicative, and the actual measured properties may vary depending on specific printing parameters (orientation, infill, temperature) [24,27,28].

### 2.3. Additive Manufacturing (FFF Printing)

A MakerBot Method X printer (MakerBot, New York, NY, USA) operating on the principle of fused deposition modeling (FDM) was used for the additive manufacturing of samples. The choice of printer was determined by technical specifications suitable for the successful processing of technical and composite materials.

The primary reason for choosing this device was its advanced heated and fully enclosed chamber system [29]. The use of PA12 CF material requires strict control of the ambient temperature, as nylon polymers are susceptible to thermal deformation and warping due to uncontrolled cooling [24,30]. The MakerBot Method X allows a constant and controlled temperature to be maintained throughout the printing process, eliminating thermal gradients and maximizing print quality and interlayer adhesion, which is particularly important for achieving reliable mechanical properties (see Table 4) [24].

Another decisive factor was the printer’s hardware compatibility with composite materials. Given that PA12 CF contains carbon fibers with high abrasive characteristics that have the potential to rapidly degrade standard brass nozzles [31], the printer was equipped with a special extrusion system. For this reason, a nozzle made of hardened steel or ruby composite was implemented, which effectively resists abrasive wear and ensures the long-term dimensional accuracy of the extruded filament, which is essential for modeling complex TPMS structures [32].

### 2.4. Three-Point Bend Testing According to ISO 178

**Figure 4:**
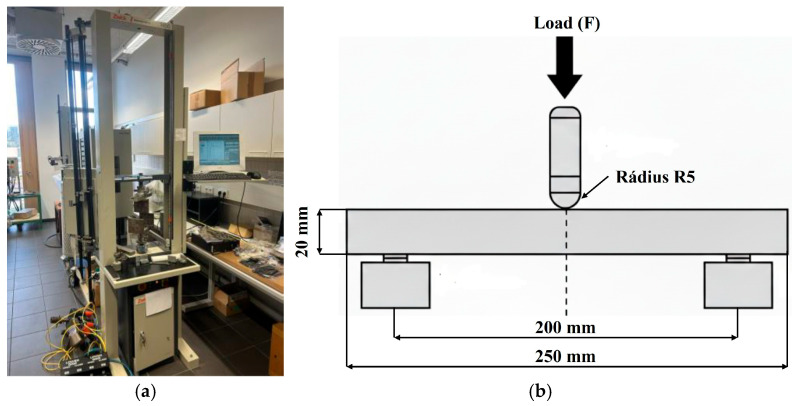
Zwick 1456 universal measuring machine (**a**) Schematic representation of the experimental setup of the three-point bending test (**b**).

A Zwick 1456 universal testing machine (Ulm, Germany) was used to perform three-point bending tests (Figure 4a). This machine is primarily designed for laboratory applications and was used to measure various mechanical properties, including tensile, bending, and compression tests [33]. The behavior of structures with uneven density under load was tested in accordance with the international standard ISO 178:2019 [34,35]. The ISO 178: 2019 standard provided us with a span-to-depth ratio that served to make deformation dominated by bending and minimize the influence of shear effects.

The testing was carried out under standardized laboratory conditions, specifically at a temperature of 20 °C and relative humidity of 50%. The key technical parameters of the Zwick 1456 testing machine include a force measurement range of up to 20 kN, which is in line with the capacity of the bending fixture according to ZwickRoell documentation [36]. The machine is equipped with a high-precision load cell (e.g., X-Force), and measurements can be controlled via TestXpert II software, which allows for load frame deformation compensation (known as “compliance correction”) for accurate evaluation of flexural modulus values [37,38].

Before the mechanical testing was carried out, the key input parameters of the bending test were precisely defined and set in accordance with ISO 178:2019. The spacing of the support elements (L) was set at 200 mm, which, given the height of the sample (h = 20 mm), ensured a span-to-depth ratio of 10:1. This setting was chosen to minimize the influence of shear stresses and ensure dominant bending stress in accordance with the recommendations for rigid polymer structures. To ensure optimal contact and minimize local damage, the radius of both support elements and the pressure pad that applied the load to the center of the specimen was set to 5 mm. Other critical parameters that were configured on the Zwick 1456 testing machine included:

- Feed rate for determining the elastic modulus: 1 mm/min;
- Test feed rate after elastic range 20 mm/min;
- Load cell capacity: The load cell used had a nominal capacity of 20 kN, which ensured sufficient range and measurement accuracy.

The mechanical properties of TPMS structures were determined using a three-point bending test at room temperature, the aim of which was to determine the maximum force (F_max_), energy absorbed until failure (E_abs_), and ductility index (µ_d_). During testing, the force curve was recorded as a function of displacement, from which the individual mechanical parameters were subsequently calculated.

### 2.5. Postprocessing and Evaluation of Bending Testing Data

After experimental testing using a three-point bend, primary data was obtained for each test sample in the form of force versus displacement (F vs. δ). This record represents a basic source of information about the bending behavior of gradient TPMS structures. From the measurements, it is possible to directly determine the maximum force (F_max_), which corresponds to the highest load value before failure. The behavior of selected cell structures during testing was evaluated in detail using energy absorption and stiffness indices. All bending tests were performed in accordance with ISO 178. For structures exhibiting progressive deformation, the absorbed energy was evaluated up to the displacement corresponding to the maximum force (F_max_), which was defined as the end point for comparison purposes.

#### 2.5.1. Energy Absorption to the Point of Fracture (E_abs_)

The energy absorbed by the sample during bending loading up to the maximum force (F_max_) is an important indicator of structural resistance and deformation stability. In this study, the absorbed energy (E_abs_) was calculated as the area under the force-displacement curve from the start of loading to the displacement corresponding to F_max_ [39]:

(1) $$E_{abs}=\int_{0}^{\delta_{max}}F\left(\delta \right)d\delta$$

where F(δ) is the applied force and δ_max_ is the displacement when the maximum force F_max_ is reached.

#### 2.5.2. Ductility Index (*μ*_*d*_)

The ductility index (µ_d_) was used to quantify the ability of TPMS structures to undergo deformation beyond the elastic regime before reaching maximum load. It was defined as the ratio between the displacement at maximum force (δ_u_ = δ_Fmax_) and the displacement at the elastic limit (δ_e_) [40]:

(2) $$\mu_{d}=\frac{\delta_{u}}{\delta_{e}}$$

Here, δ_e_ corresponds to the end of the linear elastic region determined by linear regression analysis of the initial force and displacement response, while δ_u_ represents the displacement at F_max_. This definition allows the ductility of structures exhibiting different failure modes to be evaluated on a common and objective basis.

## 3. Results

In the following subsections, we present the results of an experimental study focusing on the mechanical behavior of PA12 CF cell samples under three-point bending load. While previous research [24] has characterized in detail the basic material properties and behavior of uniform (even) TPMS structures, this study builds on these findings and focuses exclusively on the influence of gradient (uneven) material distribution. The aim is to assess how this architectural change—arising from the specific design of printed cell structures—affects their overall strength and energy absorption.

### 3.1. Analysis of Stiffness and Deformation Mechanisms

**Figure 5:**
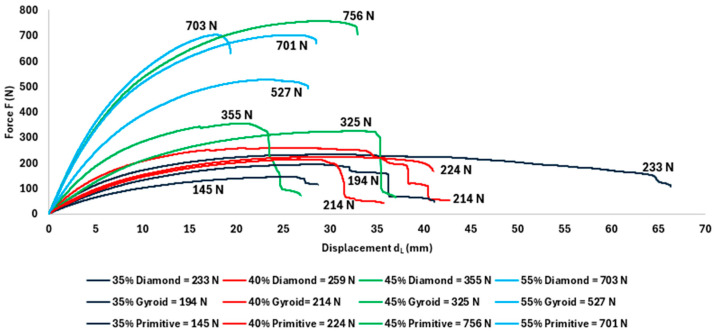
Representative Force–Strain curves of selected TPMS structures with non-uniform material distribution. The curves clearly demonstrate the contrast between strength-oriented failure (Primitive 45%) and energy-absorbing, ductile failure (Gyroid 45%).

The basic mechanical behavior of the tested TPMS structures, shown in the force-deformation curves (Figure 5), indicates significant differences in their bending response. Based on the shape of the curves, it is possible to identify different characteristics of the mechanical behavior of individual topologies at different volume fractions. Based on these curves, two dominant modes of behavior can be identified:

1. Strength-oriented behavior (Primitive):
  - Primitive samples, especially those with volume fractions of 45% and 55%, show a steep initial increase in force in the small deformation range, reflecting high initial stiffness. The highest recorded maximum force was measured for the Primitive 45% structure (F_max_ = 756 N). After reaching the maximum force, there is a significant decrease in load, accompanied by limited deformation capacity after the maximum.
2. Energy absorption-oriented behavior (Gyroid):
  - The Gyroid sample with a volume fraction of 45% exhibits a different force-deformation curve shape compared to the other tested variants. Although the maximum force reached a lower value (F_max_ = 504 N), after exceeding the elastic range, there is an extensive range of almost constant force with increasing deformation. This course leads to significantly higher absorbed energy, which reached a value of E_abs_ = 34.6 J for Gyroid 45%.

Most of the other samples tested, including Diamond and Primitive structures at lower volume fractions, exhibit transitional behavior between the extremes mentioned above, without a significantly extended region of nearly constant strength.

### 3.2. Dependence of Maximum Force (F_max_) on Volume Fraction and Type of Structure

The results of three-point bending testing of TPMS structures made of PA12 CF material show that the maximum force (F_max_) depends on the volume fraction and type of cell topology.

**Figure 6:**
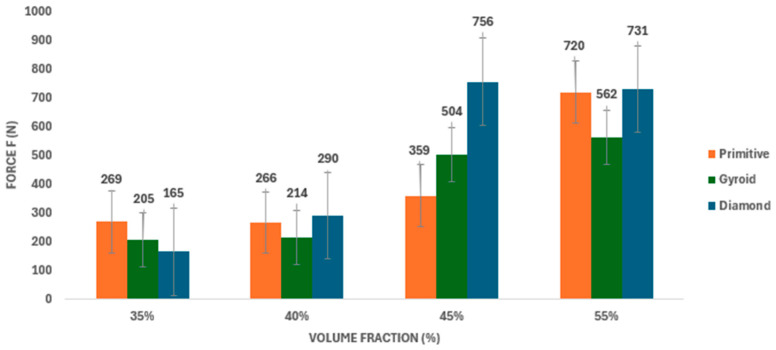
Comparison of average values of maximum force (F_max_) [N] achieved during three-point bending for TPMS structures of Primitive, Gyroid and Diamond types depending on the volume fraction of the material (35–55%).

For all three tested topologies (Diamond, Gyroid, and Primitive), an increase in F_max_ was observed with increasing volume fraction of material, as shown in Table 5 and Figure 6. The lowest maximum force values were measured at a volume fraction of 35%, while higher volume fractions led to a gradual increase in F_max_ across all structure types.

At lower volume fractions (35% and 40%), the differences between the individual topologies were relatively small. At a volume fraction of 35%, the maximum force values ranged from 165 N for the Primitive structure to 269 N for the Diamond structure.

With increasing volume share, the differences in F_max_ values between the individual topologies increased. The Primitive structure achieved the highest measured maximum force value (F_max_ = 756 N) at a volume share of 45%. At a volume share of 55%, this topology maintained a comparable level of maximum force (731 N) to the Diamond structure (720 N).

The Gyroid structure exhibited lower maximum force values compared to other topologies at volume fractions of 45% and 55%, specifically 504 N and 562 N.

Compared to the values published in previous research [24], higher maximum force values were recorded in this study, with differences being particularly evident at higher volume fractions across all TPMS topologies examined.

### 3.3. Normalization of Mechanical Response: Flexural Strength (MPa)

While the maximum force (F_max_) provides information about the load-bearing capacity of a specific tested body, for an objective comparison of the influence of topology and density, it is necessary to normalize the data to bending strength (σ_f_). This approach eliminates the influence of sample dimensions and allows direct comparison with other studies of cellular materials.

The calculation was performed according to ISO 178 using the geometric parameters of the samples (L = 200 mm, b = 20 mm, h = 20 mm). The resulting flexural strength values are summarized in Table 6.

For an objective comparison of the mechanical properties of the tested non-uniform structures, the measured maximum force values were converted to flexural strength (σ_f_). The calculation was performed in accordance with ISO 178 using the following equation:

(3) $$\sigma_{f}=\frac{3\times F_{max}\times L}{2\times b\times h^{2}}$$

where F_max_ is the maximum applied load (N), L is the distance between supports (in our case 200 mm), b is the width of the sample (20 mm), and h is the thickness of the sample (20 mm). Given the constant geometric parameters of the samples used, a constant coefficient of 0.0375 was used to calculate the strength in MPa. The resulting values are shown in Table 6.

The bending strength analysis revealed that the Diamond structure exhibits an almost linear increase in strength with volume fraction, indicating high stability of this geometry in a gradient distribution. In contrast, the Primitive structure shows an unexpected trend, where maximum strength (28.35 MPa) is achieved at a 45% fraction, while at a 55% fraction there is a slight decrease to 27.41 MPa. This phenomenon may be related to the limitations of FDM technology when printing extremely dense non-uniform walls, where local overheating of the material and the formation of internal stresses occur.

These strength results in MPa serve as an important basis for the subsequent energy absorption analysis, which will show that high strength does not always mean the best energy dissipation capacity.

### 3.4. Evaluation of Energy Absorption and Ductility Behavior

#### 3.4.1. Energy Absorption (E_abs_)

**Figure 7:**
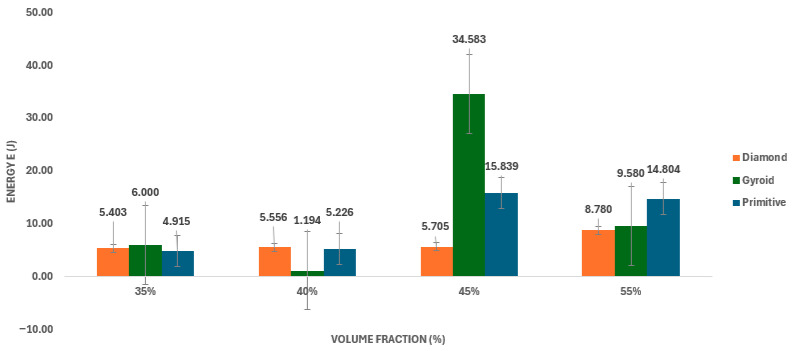
Energy absorption capacity of TPMS structures: Comparison of results (E_abs_) with indication of unexpected energy absorption of Gyroid structure at 45% volume fraction.

The absorbed energy up to the point of maximum force (E_abs_) was determined from the force-deformation curves as the area under the curve up to the value F_max_. The absorbed energy values for individual TPMS topologies at different volume fractions are shown in Table 7. The dependence of E_abs_ on volume fraction and structure type is graphically illustrated in Figure 7.

**Figure 8:**
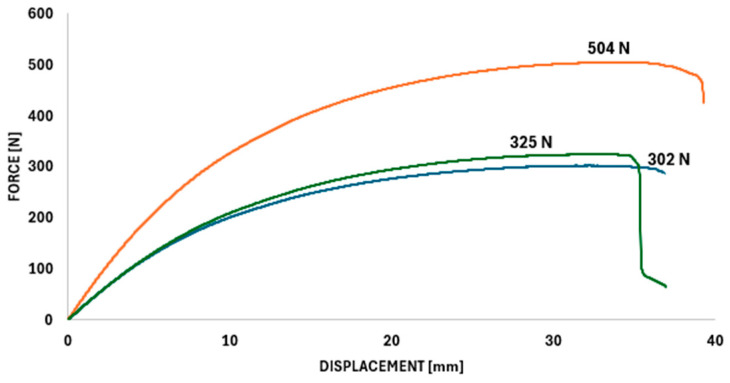
Individual force and displacement curves obtained from three-point bending tests for a gyroid structure with a nominal volume fraction of 45% (n = 3), illustrating the variability of mechanical responses and deformations.

The general trend of absorbed energy (E_abs_) growth with volume fraction is evident in the Primitive and Diamond structures (e.g., Primitive increased from 4.92 J to 14.80 J). However, the key observation concerns the Gyroid structure. At a 45% volume fraction, Gyroid achieved the maximum E_abs_ in the entire set, namely 34.58 J. This value is significantly higher than that for 55% Gyroid (9.58 J). It is assumed that this significant discrepancy is due to the influence of uneven material distribution, which at 45% led to progressive plastic deformation and an extraordinary increase in ductility instead of sudden fracture. In the Gyroid 45% configuration, a significant dispersion in the force and displacement response was observed, as shown by the individual curves in Figure 8.

#### 3.4.2. Ductility Index (µ_d_)

The ductility index (µ_d_) was calculated as the ratio of the displacement at maximum force (δ_u_) to the displacement at conventional yield (δ_u_) (see Section 2.5.2). Analysis of these values (Table 8) reveals that the Primitive structure maintained its superiority in the area of plasticity.

At low volume fractions (35% and 40%), all structures tend to have higher µ_d_ values (e.g., Diamond 35% with µ_d_ = 12.06), which is usually caused by an extremely low modulus of elasticity and low yield strength, causing even small plastic deformation to significantly exceed elastic deformation.

However, a key finding emerges at higher volume fractions. The primitive structure at 45% (µ_d_ = 8.13) and 55% (µ_d_ = 8.62) showed the highest ductility among all tested geometries. This suggests that despite its high flexural strength (which was the highest), this structure effectively uses its gradient arrangement for controlled energy dissipation through plastic deformation instead of brittle fracture. In contrast, Gyroid and Diamond at 55% volume fraction achieve µ_d_ in the range of 3.5–3.6, indicating a transition to less ductile and potentially more brittle behavior.

In all cases except Primitive 55%, the ductility of TPMS structures is relatively low. The lowest average ductility was found in the Diamond structure (average µ_d_ ≈ 2.5–3.0), while Primitive 55% with a value of µ_d_ = 8.62 indicates the highest ability to dissipate energy through controlled deformation before failure. This correlates with its high maximum force (F_max_), suggesting that although the Primitive structure effectively carries the load, it is also able to maintain a relatively high degree of plasticity due to its spatial arrangement.

## 4. Discussion

The discussion focuses on the interpretation of measured values of maximum force (F_max_), flexural strength (σ_f_), absorbed energy (E_abs_), and ductility index (µ_d_) from the perspective of flexural mechanics and effective load transfer in TPMS structures with gradient material distribution. The introduction of normalized flexural strength in MPa, calculated according to ISO 178, allows for a more objective assessment of the mechanical response independent of the geometric dimensions of the samples. The experimental results are compared with the behavior of standard uniform (uniform) TPMS structures described in the existing literature [24], with an emphasis on identifying the mechanisms by which the gradient distribution of material affects the deformation response and load-bearing degradation processes after reaching maximum force. This approach directly responds to the need to quantify the contribution of non-uniform architecture compared to conventional grid structures. However, uniform reference structures were not experimentally tested in this study, and therefore the comparison assumes comparable material, manufacturing, and testing conditions.

### 4.1. Effect of Volume Fraction on Effective Mechanical Response

Our research confirms that increasing the volume fraction of the material leads to an increase in the mechanical resistance of TPMS structures, but the most significant qualitative leap in mechanical response was observed when switching from a 40% to a 45% volume fraction in the Primitive structure. The Primitive structure with a 45% volume fraction achieved a maximum bending force of F_max_ = 756 N, which corresponds to a bending strength of σ_f_ = 28.35 MPa. This value represents the highest strength achieved in the entire experimental set, significantly exceeding even the values of the Gyroid structure at a higher volume fraction of 55% (F_max_ = 562 N; σ_f_ = 21.08 MPa).

From the point of view of bending mechanics, this phenomenon can be explained by more efficient use of material in areas with higher bending stress. In gradient TPMS structures, the material is concentrated towards areas further away from the neutral axis of bending, resulting in an increase in the second moment of area with a relatively small increase in weight. This effect leads to higher bending stiffness and a delay in the onset of local load-bearing degradation in critical areas of the structure. It is important to note that in the Primitive structure, increasing the volume fraction to 55% resulted in a slight decrease in strength to 27.41 MPa, indicating the existence of a limiting density beyond which the gradient distribution of material does not further increase the static load capacity.

The strength value achieved for the Primitive 45% structure significantly exceeds the maximum forces recorded for uniform Primitive structures made of the same PA12 CF material, which were published in the literature [24] and reached approximately 160 N (σ_f_ = 6.30 MPa) at a volume fraction of 30%. Although the volume fractions are not identical, the comparison remains relevant as both studies use the same material, manufacturing technology (FDM), and three-point bending configuration. The results suggest that the gradient distribution of the material not only leads to a linear scaling of the mechanical response with density, but also fundamentally changes the load transfer mechanism and the resistance of the structure to load-bearing degradation after reaching the maximum force (F_max_).

The Primitive structure, characterized by an orthogonal arrangement of load-bearing elements, exhibits behavior similar to a beam-dominated deformation mechanism, which allows for more efficient transfer of bending moment and greater stability under load. However, at a volume fraction of 55%, there is only a slight increase in F_max_ compared to the Diamond structure (720 N and 27 MPa, respectively), indicating an approaching limit of efficiency for further density increases given the gradient profile used.

### 4.2. Analysis of the Gyroid 45% Anomaly and Ductility Behavior

One of the observed deviations from the overall trend of experimental results is the high value of absorbed energy in the Gyroid structure at a 45% volume fraction (E_abs_ = 34.58 J). This value is several times higher than the absorbed energy typically measured in uniform TPMS structures made of PA12 CF, which are characterized in the literature mainly by a sudden drop in load-bearing capacity after reaching maximum force and limited plastic deformation [24]. Compared to a uniform Gyroid (35% share), which achieves a strength of only approximately 4.35 MPa [24], our gradient Gyroid 45% not only exhibits higher strength (18.90 MPa), but above all a fundamentally different deformation mode. However, given the limited number of samples tested (n = 3), this result should be interpreted with appropriate caution.

From a mechanical point of view, this phenomenon does not correlate directly with the maximum force (F_max_ = 504 N; σ_f_ = 18.90 MPa) but is associated with a different deformation mechanism. Gyroid topology is characterized by smooth, curved geometry without sharp transitions, which promotes stress redistribution and suppresses the localization of load-bearing degradation. In combination with a gradient wall thickness distribution, this can, under certain conditions, lead to sequential and progressive degradation of the load-bearing capacity of individual parts of the structure, rather than a sudden global decrease in load-bearing capacity. This mechanism of “sequential folding” allows the structure to maintain a high level of stress over a long deflection path, which explains the extreme increase in energy capacity despite the fact that it is not the strongest sample in the set.

Although the ductility index value for Gyroid 45% (µ_d_ = 4.70) was not the highest among the tested variants, the deformation path was exceptionally long and stable, which was reflected in a significant plastic plateau on the force-deflection curve, as can be seen in Figure 8. This shows the individual force and displacement curves obtained for the gyroid structure with a nominal volume fraction of 45%, revealing a noticeable dispersion in the mechanical response. This type of behavior is typical for energy-absorbing materials and indicates the potential sensitivity of gyroid topology to local inhomogeneities caused by additive manufacturing, although further experimental verification would be necessary to confirm the reproducibility of this effect.

### 4.3. Optimization with Respect to Gradient Distribution

Na Based on a comprehensive evaluation of the parameters F_max_, E_abs_, and µ_d_, it can be concluded that the Primitive structure with volume fractions of 45% and 55% represents the most balanced compromise between strength and ductility. High ductility index values (up to µ_d_ = 8.62) combined with high maximum strength and flexural strength (up to 28.35 MPa) indicate that the cubic arrangement of Primitive topology enables effective distribution of bending loads and slows down the initiation and development of local areas of load-bearing degradation, even in the presence of inhomogeneities caused by the FDM process. This finding is crucial because it shows that gradient design can compensate for the geometric complexity of the structure and ensure stability even under high stress levels.

From the point of view of practical applications, Primitive structure therefore appears to be the most resistant to the negative effects of uneven material distribution on mechanical response in bending, which are an inherent part of additive manufacturing.

The gradient approach thus represents a promising tool for designing optimized TPMS structures exposed to bending loads, although further experimental studies involving direct comparison with uniform reference samples at identical MPa values are needed to clearly quantify its benefits.

It should be emphasized that the volume fractions given represent nominal values defined in the CAD model; the mechanical response of the samples therefore reflects the combined effect of the designed gradient and the inevitable local deviations caused by the FDM process. However, it is precisely the normalization of results for bending strength (σ_f_) that provides a more reliable basis for future optimization of these metamaterials, as it eliminates dimensional effects and focuses on the internal efficiency of the proposed architecture.

## 5. Conclusions

This research focused on analyzing the mechanical behavior of three types of TPMS structures (Primitive, Gyroid, and Diamond) with an applied linear gradient of local density in the range of total volume fractions from 35% to 55%. Experimental data from three-point bending tests performed according to ISO 178 confirm that the synergy between cell topology and gradient material distribution is critical for defining strength and absorption capacity. The introduction of normalized flexural strength (σ_f_) has enabled the exact quantification of this contribution, with the tested samples achieving values ranging from 6.19 MPa to 28.35 MPa depending on topology and density.

Compared to uniform TPMS structures made of PA12 CF (according to data from a previous study [24]), and corresponding flexural strength (σ_f_). For example, in the Primitive structure (35% volume fraction), there was an increase in strength from approximately 168 N in the uniform arrangement to 165 N in the gradient, but at higher volume fractions (55%), the gradient structure dramatically outperformed the uniform structure (731 N vs. 314 N). This striking difference confirms the effectiveness of gradient design as a highly effective optimization strategy for increasing the specific stiffness and load-bearing capacity of cells.

The main findings can be summarized as follows:

1. Dominance of the Primitive structure in strength: When comparing the tested topologies, the Primitive structure proved to be the most mechanically resistant. The Primitive variant, with a total share of 45%, achieved the highest maximum force (F_max_ = 756 N), which corresponds to a peak bending strength of σ_f_ = 28.35 MPa. The robust connection of the walls in this topology allows for more efficient transfer of bending loads compared to the Gyroid and Diamond structures with an identical gradient, with strength stabilizing at 27.41 MPa at a 55% share.
2. Optimal toughness (ductility index): The Primitive structure with volume fractions of 45% and 55% demonstrated not only high normalized strength but also the highest plastic deformation capacity. With a ductility index (µ_d_) reaching a value of up to 8.62 (in accordance with Table 8), this variant is an optimal candidate for applications requiring predictable failure without sudden collapse, while maintaining integrity even at high levels of bending stress.
3. Anomalous deformation behavior of the Gyroid structure: A key finding is the anomalously high absorbed energy for the Gyroid 45% variant (34.58 J), which surpassed even the sample with a higher proportion of 55%. This phenomenon suggests that, with a specific combination of Gyroid topology and gradient, the deformation mechanism is stabilized through the gradual collapse of cells, which maximizes the area under the force-displacement curve despite lower peak strength (18.90 MPa) compared to other structures.
4. Functional specialization of topologies: The results confirm that the choice of TPMS geometry directly determines its mechanical purpose. While Primitive excels in applications focused on static strength (MPa), Gyroid demonstrates greater potential for controlled energy absorption. The Diamond structure represents a balanced solution with high stability and a linear increase in mechanical response with increasing density, which is important for predictable engineering design.

In terms of flexural load and resistance to the inherent unevenness of 3D printing (PA12 CF), the most effective solution is the Primitive structure with an average volume fraction of 45–55%, which achieves a peak flexural strength of up to 28.35 MPa. The implementation of a gradient material distribution allows for local adaptation of the mechanical response, which represents a significant advance for the design of lightweight structures in aviation, the automotive industry, and biomechanics. This approach, based on calculations according to ISO 178, has shown that targeted redistribution of material in critical bending zones dramatically increases the efficiency of composite material use compared to conventional uniform topologies.

Recommendations for future research

For a comprehensive understanding of the behavior of gradient TPMS structures, future research should focus on:

- Dynamic testing: Examination of structural behavior through impact tests to simulate high-speed loading conditions, thereby verifying the validity of static flexural strength values (σ_f_) under dynamic conditions.
- Optimization of transition zones: Deeper investigation of the influence of different types of gradient transitions (e.g., nonlinear) between volume fractions to achieve even greater uniformity of the deformation curve and maximize energy absorption without the risk of local stress concentrations.
- Impact of production parameters: A more detailed analysis of the impact of print orientation and thermal history in FDM technology on the resulting flexural strength to eliminate undesirable anomalies observed at higher material densities.
- Application extension: Application of the determined optimal geometric and gradient parameters to other types of materials (e.g., metals produced by SLM technology) and other manufacturing technologies in order to generalize the model for a wider range of engineering applications in accordance with international standards for testing materials.

## Figures and Tables

**Table 1:** Selected TPMS structures and their mathematical expression [24].

**TPMS Structure**	**Mathematical Expression**
Schwarz Diamond 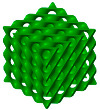	f_(x, y, z)_ = sin (x)·sin (y)·sin (z) + sin (x)·cos (y)·cos (z) + cos (x)·sin (y)·cos (z) + cos (x)·cos (y)·sin (z) = t
Schoen Gyroid 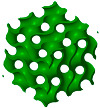	f_(x, y, z)_ = sin (x)·cos (y) + sin (y)·cos (z) + sin (z)·cos (x) = t
Schwarz Primitive 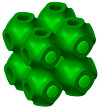	f_(x, y, z)_ = cos (x) + cos (y) + cos (z) = t

**Table 2:** FDM printing parameters [24].

Parameters	Value
Material	PA12 CF
Filament diameter	1.75 mm
Printer	MakerBot Method X
Nozzle Diameter	0.4 mm
Layer height	0.2 mm
Line width	0.45 mm
Temperature in the chamber	65 °C
Pad temperature	65 °C
Printing speed	35 mm/s
Printing strategy	Default
Printing orientation	Longitudinal axis parallel to build plate

**Table 3:** Properties of the material Nylon 12 CF [24].

Features	
Density	1.04 g/cm^3^
Melting point	180–190 °C
Melting resistance (continuous)	−40 °C to 120 °C
Melting resistance (short-term)	180 °C
Tensile modulus	10 GPa
Tensile strength	83.5 MPa
Elongation at break	10%
Toughness	50 kJ/m^2^

**Table 4:** MakerBot Method X printer specifications [24].

Specification	
Technology	FDM
Dimensional accuracy	±0.1 mm
Print speed	up to 100 mm/s
Press room	250 × 250 × 250 mm
Maximum print chamber temperature	100 °C
Maximum print head temperature	300 °C
Printer dimensions	520 × 520 × 720 mm
Printer weight	35 kg
Number of print heads	2
Custom Cloud and Slicer for post-process model creation	YES

**Table 5:** Influence of cell geometry on maximum force (F_max_) within TPMS structures at different volume fraction levels compared to uniform material distribution [24].

Volume Fraction	Design	Diamond F_max_ (N)	Gyroid F_max_ (N)	Primitive F_max_ (N)	Reference
35%	Gradient	269 ± 30.20	205 ± 6.35	165 ± 16.17	This study
	Uniform	160	116	168	[24]
40%	Gradient	266 ± 11.24	214 ± 5.51	290 ± 42.36	This study
	Uniform	213	252	151	[24]
45%	Gradient	359 ± 8.89	504 ± 110.58	756 ± 42.74	This study
	Uniform	279	242	179	[24]
55%	Gradient	720 ± 15.52	562 ± 35.00	731 ± 20.03	This study
	Uniform	307	283	314	[24]

**Table 6:** Calculated flexural strength (σ_f_) for different TPMS topologies and volume fractions.

Volume Fraction	Diamond σ_f_ (MPa)	Gyroid σ_f_ (MPa)	Primitive σ_f_ (MPa)
35%	10.09 ± 1.13	7.69 ± 0.24	6.19 ± 0.61
40%	9.98 ± 0.42	8.03 ± 0.21	10.88 ± 1.59
45%	13.46 ± 0.33	18.90 ± 4.15	28.35 ± 1.60
55%	27.00 ± 0.58	21.08 ± 1.31	27.41 ± 0.75

**Table 7:** Energy absorption (E_abs_) in J to the point of maximum force.

Volume Fraction	Diamond E_abs_ (J)	Gyroid E_abs_ (J)	Primitive E_abs_ (J)
35%	5.4	6.00	4.92
40%	5.56	1.19	5.23
45%	5.71	34.58	15.84
55%	8.78	9.58	14.80

**Table 8:** Average values of Ductility Index (*µ_d_*).

Volume Fraction	Diamond µ_d_	Gyroid µ_d_	Primitive µ_d_
35%	12.06	7.96	5.37
40%	6.43	3.14	5.06
45%	5.09	4.70	8.13
55%	3.51	3.58	8.62

## Data Availability

The original contributions presented in the study are included in the article, and further inquiries can be directed to the corresponding author.

## Abbreviations

The following abbreviations are used in this manuscript:

TPMS	Triple Periodic Minimal Surface
FDM	Fused Deposition Modeling
